# RpoS Regulates a Novel Type of Plasmid DNA Transfer in *Escherichia coli*

**DOI:** 10.1371/journal.pone.0033514

**Published:** 2012-03-16

**Authors:** Yanmei Zhang, Chunyu Shi, Jiafei Yu, Jingjing Ren, Dongchang Sun

**Affiliations:** 1 State Key Laboratory Breeding Base for Zhejiang Sustainable Pest and Disease Control, Zhejiang Academy of Agricultural Sciences, Hangzhou, P. R. China; 2 Institute of Plant Protection and Micriobiology, Zhejiang Academy of Agricultural Sciences, Hangzhou, P. R. China; 3 Department of Pathology, University of Virginia, Charlottesville, Virginia, United States of America; Université Paris Descartes; INSERM, U1002., France

## Abstract

Spontaneous plasmid transformation of *Escherichia coli* is independent of the DNA uptake machinery for single-stranded DNA (ssDNA) entry. The one-hit kinetic pattern of plasmid transformation indicates that double-stranded DNA (dsDNA) enters *E. coli* cells on agar plates. However, DNA uptake and transformation regulation remain unclear in this new type of plasmid transformation. In this study, we developed our previous plasmid transformation system and induced competence at early stationary phase. Despite of inoculum size, the development of competence was determined by optical cell density. DNase I interruption experiment showed that DNA was taken up exponentially within the initial 2 minutes and most transforming DNA entered *E. coli* cells within 10 minutes on LB-agar plates. A half-order kinetics between recipient cells and transformants was identified when cell density was high on plates. To determine whether the stationary phase master regulator RpoS plays roles in plasmid transformation, we investigated the effects of inactivating and over-expressing its encoding gene *rpoS* on plasmid transformation. The inactivation of *rpoS* systematically reduced transformation frequency, while over-expressing *rpoS* increased plasmid transformation. Normally, RpoS recognizes promoters by its lysine 173 (K173). We found that the K173E mutation caused RpoS unable to promote plasmid transformation, further confirming a role of RpoS in regulating plasmid transformation. In classical transformation, DNA was transferred across membranes by DNA uptake proteins and integrated by DNA processing proteins. At stationary growth phase, RpoS regulates some genes encoding membrane/periplasmic proteins and DNA processing proteins. We quantified transcription of 22 of them and found that transcription of only 4 genes (*osmC*, *yqjC*, *ygiW* and *ugpC*) encoding membrane/periplasmic proteins showed significant differential expression when wildtype RpoS and RpoS^K173E^ mutant were expressed. Further investigation showed that inactivation of any one of these genes did not significantly reduce transformation, suggesting that RpoS may regulate plasmid transformation through other/multiple target genes.

## Introduction

Gene transfer through plasmid conjugation or transformation is one of the major cause of antibiotic resistance in bacteria [1]. The emergency of many life killing superbugs, such as NDM-1 bacteria and enterohaemorrhagic *Escherichia coli* (EHEC), is often a consequence of the transfer of antibiotic resistance genes mediated by plasmids [2], [3]. Although plasmid conjugation was found in *E. coli* long ago, this species has traditionally been considered not to be naturally transformable because it is transformable only after special treatments (i.e. electric shock or Ca^2+^ stimulation and heat shock) [4]. While a complete set of competence gene homologs for the assembly of a conserved DNA uptake machinery were found in the genome of *E. coli* [5]. Moreover, transcription of some competence genes is inducible by a competence regulator homolog Sxy in *E. coli* [6]. Our work, together with the work from several other groups, showed that *E. coli* is able to acquire naked plasmid DNA on agar plates at 37°C without the addition of Ca^2+^ or heat shock [7], [8], [9]. Our further investigation revealed that plasmid transformation on plates is promoted by agar/agarose, a stimulation that is unrelated to divalent cations like Ca^2+^ , Mg^2+^ and Mn^2+^ [10]. Interestingly, none of the DNA uptake gene homologs were found to be involved in mediating spontaneous plasmid transformation of *E. coli* [10]. The dose-response curve of transformation frequency as a function of DNA concentration showed that *E. coli* cells acquired plasmid DNA with a single hit kinetics, suggesting that plasmid DNA enters *E. coli* cells through a different route which allows double-stranded DNA (dsDNA) entry [10]. Entry of dsDNA in *E. coli* is different from that in other naturally transformable bacteria, which often use the DNA uptake machinery for single-stranded DNA (ssDNA) uptake [11] and DNA binding proteins for processing and integrating the incoming ssDNA [12]. For example, in plasmid transformation of *Streptococcus pneumoniae*, two strands of ssDNA from two plasmid molecules are taken up into the cytoplasm to re-establish a new plasmid with the assistance from recombinase RecA in the cytoplasm [13].

Our previous work and others showed that *E. coli* develops competence for spontaneous plasmid DNA uptake at stationary phase [8], [9]. RpoS is an alternative sigma factor which is induced at stationary phase or under conditions of starvation or stress (e.g. temperature, osmolarity or pH) [14], [15]. Whole-genome microarray data reveal that more than 480 genes are potentially regulated by RpoS under different stress conditions [16], [17], [18], [19]. At 37°C, RpoS is degraded by the protease in the exponential growth phase but protected from protease degradation at stationary phase [14], [15]. When *E. coli* was incubated at a temperature lower than 30°C, RpoS begins accumulating at the exponential phase because its translation is highly promoted by a small RNA DsrA [20]. It remains unknown whether RpoS, the stationary phase master regulator, plays any roles in plasmid transformation of *E. coli*.

In this study, we further documented natural competence for plasmid transformation in a developed plasmid transformation system. With this transformation system, we first studied the development of competence and examined the effect of inoculum sizes on plasmid transformation. Next, we investigated DNA uptake kinetics and the effect of cell density on plasmid transformation on LB-agar plates. The potential involvement of RpoS in spontaneous plasmid transformation was examined by gene inactivation and complementation, in addition to the effect of a single amino acid in RpoS, lysine 173, which is thought to be important to RpoS selectivity. Finally, to search for new candidates which may be involved in spontaneous plasmid transformation of *E. coli*, we compared transcription patterns of a series of RpoS-regulated genes in *rpoS*+ and *rpoS−* strains through Real-Time PCR (RT-PCR) and examined their potential roles in plasmid transformation.

## Results

### 1. The development of competence for plasmid transformation

Spontaneous plasmid transformation on agar plates has been documented at 37°C [8], [9], [10]. In our previous study, we established a novel transformation system to show that *E. coli* is naturally transformable by treating cells with static culture in a beaker [8], [10]. To further explore spontaneous plasmid transformation in *E. coli*, we developed the plasmid transformation system at 30°C, a common temperature in our environment, and static culture was omitted. This transformation system did not require heat shock or the addition of Ca^2+^. In this developed transformation system, we investigated competence development in wild-type *E. coli* K-12 strains MC4100 (kindly donated by Dr. Regine Hengge-Aronis) and BW25113 [21] and their derivatives.

**Figure 1:**
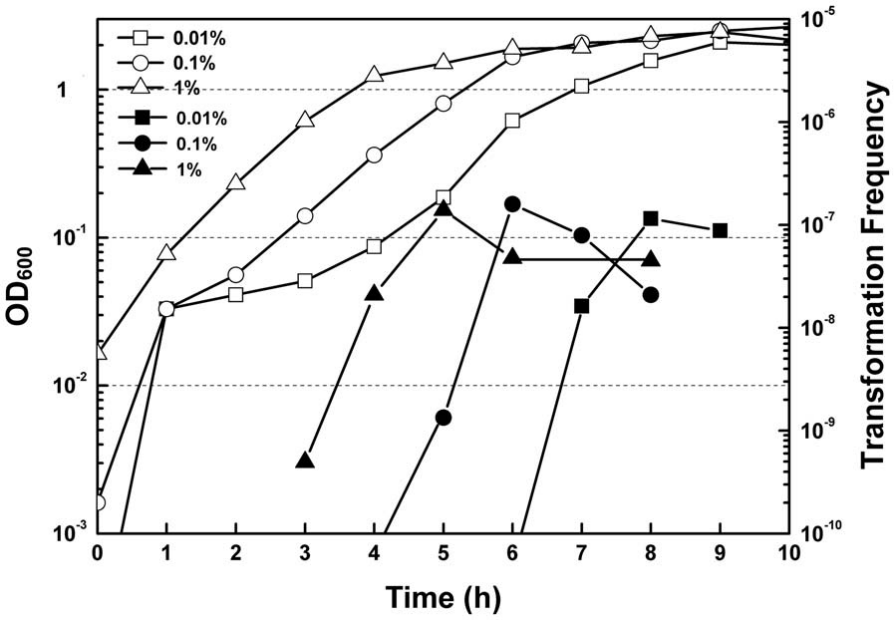
Effect of inoculum size on plasmid transformation. Overnight grown (13 hr) *E. coli* MC4100 was inoculated into three triangle flasks each containing 100 ml of fresh LB at ratios of 1∶100 (triangle), 1∶1, 000 (circle) and 1∶100, 000 (rectangle). During incubation, OD_600_ of the cultures was measured periodically (open symbols). Transformation was performed at intervals as described in Materials and Methods and transformation frequencies were shown (solid symbols). Representative data from three independent experiments were shown.

To know competence development during cell growth, we examined transformation patterns as a function of culture time with different inoculum sizes. To prepare recipient cells for plasmid transformation, overnight grown culture in LB broth was inoculated to 100 ml of 1.5× LB (containing yeast extract 7.5 g/L, tryptone 15 g/L and NaCl 7.5 g/L) with a ratio of 1∶100, 1∶1, 000 and 1∶ 10, 000 followed by incubation at 30°C with a low speed (150 rpm). At intervals, 500 µl of the culture was recovered by centrifugation and 450 µl of the supernatant was discarded. Cell pellets were resuspended in the remaining 50 µl supernatant with the addition of plasmid DNA. Transformation was performed by plating the above mixture onto selective plates containing 5% agar. We observed that few exponentially growing *E. coli* cells were transformed despite of the inoculum sizes (Figure 1). This is in contrast to artificial transformation which requires exponentially growing recipient cells. Instead, competence began to develop at the transition of exponential phase to stationary phase, reached the maximum at the entry of stationary phase and then moderately decreased (Figure 1). Competence development with three different inoculum sizes consistently showed that the highest transformation frequency (1∼2×10^−7^) occurred when the optical density at 600 nanometer (OD_600_) reached ∼1.5, no matter how long the cells were cultured (Figure 1). In the following experiments, we investigated plasmid transformation when cells grew to an OD_600_ of ∼1.5 unless otherwise indicated.

### 2. The kinetics of DNA uptake during plasmid transformation on plates

**Figure 2:**
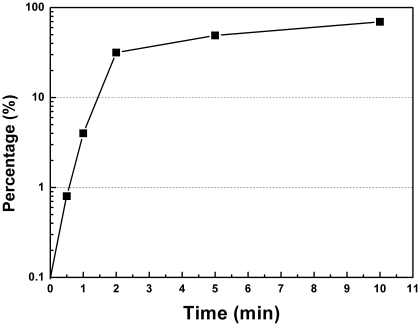
Kinetics of DNA entry on LB-agar plates. *E. coli* MC4100 cell pellets from the culture at OD_600_ of 1.5 were resuspended in the supernatant and mixed with plasmid pDsRED at a concentration of 67 µg/ml. The liquid mixture was plated on selective LB-agar plates (50 µl per plate). At intervals, excessive DNase I was spread on one of these plates. The sample without DNase I treatment was set as the control. Percentages of remaining transformants (which indicates DNA uptake kinetics) were calculated by dividing the number of transformants with DNase I treatment by the number of transformants without DNase I treatment. Representative data from three independent experiments were shown.

Our previous study and the others showed that plasmid transformation of *E. coli* occurred on agar plates at 37°C [7], [8], [9]. To test whether DNA uptake occurred prior to plating, excessive DNase I was added after co-incubation of cells from the culture of OD_600_ of 1.5 and pDsRED in the liquid culture. For both MC4100 and BW25113, no transformants were detected (Table 1). To know when DNA entered competent cells on plates, immediately after plating the mixture of *E. coli* culture and plasmid DNA, excessive DNase I was spread on the LB-agar plates. No transformants were detected in repeated experiments (n>3). This result indicates that plasmid transformation should occur on LB-agar plates after plating. To explore the kinetics of DNA uptake on agar plates, following the plating of the mixture of competent cells and plasmid DNA, we interrupted DNA uptake by spreading excessive DNase I on LB-agar plates at intervals. We found that DNA was taken up exponentially after spreading competent cells and plasmid DNA on the surface of LB-agar plates. At the start time point (0 minute), no transformants were detected (Figure 2), implying that DNA had not entered cells at that time. Within the first 2 minutes, more than one third of the competent cells acquired plasmid DNA which was not sensitive to DNase I degradation (Figure 2). Five minutes later, the addition of DNase I reduced transformation frequency less than a half (Figure 2). Within the initial 10 minutes on agar plates, 71.9% of the transforming plasmid DNA entered the DNase I insensitive state (Figure 2). The rapid uptake of DNA implies that the route for dsDNA entry might be assembled in the liquid culture before plating but quickly activated on agar plates. Alternatively, stresses introduced by exposure of planktonic cells on plates may contribute to DNA entry. We investigated potential effects of physical stress by plating and oxidative/anti-oxidative stress on plasmid transformation and failed to detect obvious change of transformation frequency by these stresses (see Discussion S1).

**Table 1:** Effect of DNase I in liquid culture before plating.

	10 min		20 min	
	+DNase I	−DNase I	+DNase I	−DNase I
MC4100 Tf*	0	1.0×10^−6^±1.4×10^−7^	0	5.6×10^−7^±9.3×10^−8^
BW25113 Tf	0	1.7×10^−6^±5.2×10^−8^	0	3.5×10^−6^±2.0×10^−7^

* Tf: Transformation frequency.

### 3. Effect of high cell density on plasmid transformation

**Figure 3:**
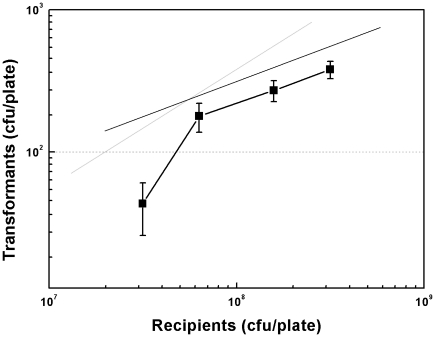
Effect of cell density on plasmid transformation. *E. coli* MC4100 cell pellets from 100 µl, 200 µl, 500 µl and 1 ml of the culture at OD_600_ of 1.5 were transformed as described in Materials and Methods. The experiment was performed in duplicate. Each point denotes an average of 4 samples. Error bars denote standard deviation. Gray and black lines indicate slopes of 1 and 0.5, respectively.

In our previous study, we examined the effect of cell density by serially diluting static culture and observed a non linear relationship between cell density and transformation frequency at low cell density (1.41×10^5^∼3.53×10^5^ CFU/cm^2^) on medium size plates (diameter of 8.5 cm) [10]. In this study, we investigated the effect of a higher cell density by concentration (with a method described in the Materials and Methods section) and plated competent *E. coli* cells on smaller agar plates (diameter of 6 cm). When the cell density was concentrated 2-fold, from 3.16×10^7^ CFU/plate (1.12×10^6^ CFU/cm^2^) to 6.31×10^7^ CFU/plate (2.23×10^6^ CFU/cm^2^), transformation frequency was increased 4-fold (Figure 3). Surprisingly, when cell density was further increased from 6.31×10^7^ CFU/plate (2.23×10^6^ CFU/cm^2^) to 3.16×10^8^ CFU/plate (1.12×10^7^ CFU/cm^2^), a half-order kinetics between recipient cells and transformants was observed (Figure 3).

### 4. RpoS regulates plasmid transformation

**Figure 4:**
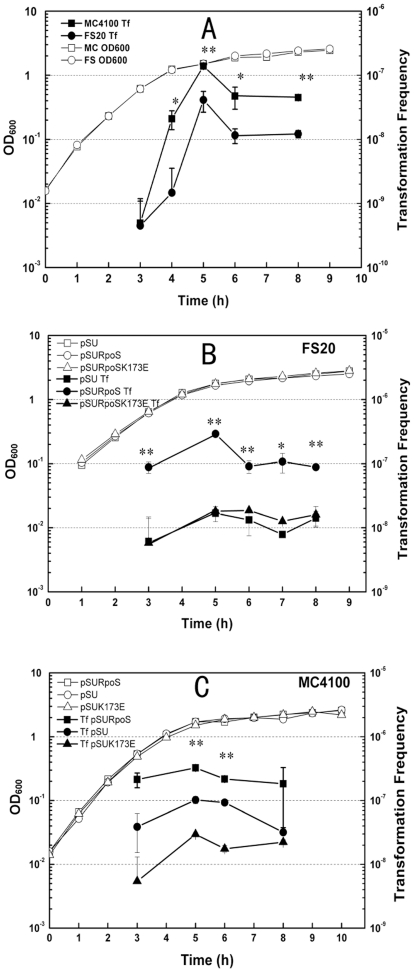
Plasmid transformation regulated by RpoS. During 3∼8 hours of incubation at 30°C, at intervals, transformation frequencies (solid symbols) and optimal densities of the cultures (open symbols) of MC4100 (*rpoS*+) and FS20 (*rpoS*−) (A); FS20-pSU, FS20-pSURpoS, FS20-pSURpoSK173E (B); and MC4100-pSU, MC4100-pSURpoS, MC4100-pSURpoSK173E (C) were measured. Transformation was performed as described in Materials and Methods. Each point denotes an average of 4 samples. Error bars denote standard deviation. ^*^ P value ≤ 0.05; ^**^ P value ≤0.01.

Two independent groups reported spontaneous plasmid transformation at stationary phase [8], [9]. In this study, we again observed that competence was induced at the transition of exponential phase to stationary phase at 30°C (Figure 1). RpoS, an alternative sigma factor [14], [15], activates the transcription of many stress induced genes, including genes encoding membrane and periplasmic proteins and DNA processing genes [16], [17], [18], [19]. In the classical transformation model, membrane proteins and DNA processing proteins are required for DNA uptake and integration [11]. As plasmid transformation of *E. coli* does not require components of the machinery for classical DNA uptake [9], [10], we questioned whether a new set of DNA uptake and processing genes are involved in mediating plasmid dsDNA transfer and if the stationary phase master regulator RpoS regulates transcription of some components of this set of genes. We first investigated the latter possibility. To check whether RpoS was involved, we compared transformation frequencies between MC4100 and its *rpoS* mutant derivative FS20 (Table 2). The growth curve patterns of the two strains were almost identical (Figure 4A). Neither did we observe that the inactivation of *rpoS* had obvious effect on viability during cell growth (data not shown). However, transformation frequency of MC4100 was more than 10-fold higher than FS20 after 4 hours of incubation (OD_600_, ∼1.0) (Figure 4A). After 5, 6 and 8 hours of incubation, transformation frequencies of MC4100 were systematically more than 3-fold of that of FS20 (Figure 4A). The effect of *rpoS* on plasmid transformation of *E. coli* was more evident by over-expressing this gene in an *rpoS* mutant FS20. At all examined time points (during 3 to 8 hours of incubation), compared with FS20 carrying an empty vector, transformation frequencies were systematically increased ∼10 fold by over-expressing *rpoS* with pSURpoS (Figure 4B), a plasmid which has ∼10 copies per cell. Additionally, over-expressing *rpoS* on the plasmid pSURpoS also increased transformation frequency 2.33 to 5.73 folds during the growth of the wild type strain MC4100 (Figure 4C).These data show that *rpoS* is involved in promotion of plasmid transformation of *E. coli*.

**Table 2:** Strains and plasmids used in this study.

*E. coli* strain or plasmid	Relevant genotype^a^ and/or description	Source or reference
*E. coli* strains		
ZK126	W3110 *ΔlacU169 tna-2*	[34]
MC4100	F- λ- *araD139 Δ(argF-lac)U169 rpsL150 relA deoC1 ptsF25 rbsR flbB5301*	Regine Hengge-Aronis
FS20	MC4100::*ΔrpoS*::*kan*; Kan^r^	Regine Hengge-Aronis
FS20-pSU	FS20 containing pSU, Cm^r^	This study
FS20-pSURpoS	FS20 containing pSURpoS, Cm^r^	This study
FS20-pSURpoS^K173E^	FS20 containing pSURpoS^K173E^, Cm^r^	This study
MC4100-pSU	FS20 containing pSU, Cm^r^	This study
MC4100-pSURpoS	FS20 containing pSURpoS, Cm^r^	This study
MC4100-pSURpoS^K173E^	FS20 containing pSURpoS^K173E^, Cm^r^	This study
BW25113	F- λ- Δ*(araD-araB)567* Δ*lacZ4787*(::rrnB-3) *rph-1 hsdR514* Δ*(rhaD-rhaB)568*	[21]
JW1477	BW25113::*ΔosmC*::*kan*	[21]
JW2992	BW25113::*ΔygiW*::*kan*	[21]
JW5516	BW25113::*ΔyqjC*::*kan*	[21]
JW3415	BW25113::*ΔugpC*::*kan*	[21]
JW5437	BW25113::*ΔrpoS*::*kan*	[21]
Plasmids		
pCHAP3100	p15A replicon; pSU18 carrying *ppdD*; Cm^r^	[35]
pDsRED	pUC replicon; red fluorescence protein-expressing plasmid; Amp^r^	[8]
pUCRpoS	pUC18 derivative; carrying an *rpoS* gene; Amp^r^	This study
pSURpoS	pCHAP3100 derivative; carrying an *rpoS* gene; Cm^r^	This study
pSU	pSURpoS with *rpoS* deletion; Cm^r^	This study
pSURpoS^K173E^	pSURpoS with K173E mutation; Cm^r^	This study

a Cm^r^, chloramphenicol resistance; Amp^r^, ampicillin resistance; Kan^r^, kanamycin resistance.

It has been documented that RpoS recognizes promoters through K173 and the mutation of K173 to glutamic acid 173 (K173E) makes RpoS unable to initiate the transcription of targeted genes [22]. To further confirm a role of RpoS on plasmid transformation, we examined the effect of K173E mutation on plasmid transformation. We found that transformation frequencies of FS20-pSURpoS^K173E^ were ∼10-fold lower than that of FS20-pSURpoS and almost identical to that of FS20 carrying an empty vector at all examined time points during cell growth (Figure 4B). Interestingly, over-expressing RpoS^K173E^ reduced the transformation frequency of MC4100 to a level similar to that of FS20 (Figure 4C), showing a dominant effect of RpoS^K173E^ on plasmid transformation. By contrast, the dominant effect of RpoS^K173E^ on plasmid transformation of FS20 was not obvious under such conditions (Figure 4B). Together, we conclude that RpoS is involved in regulating plasmid transformation of *E. coli*.

### 5. RpoS-regulated genes and plasmid transformation

**Figure 5:**
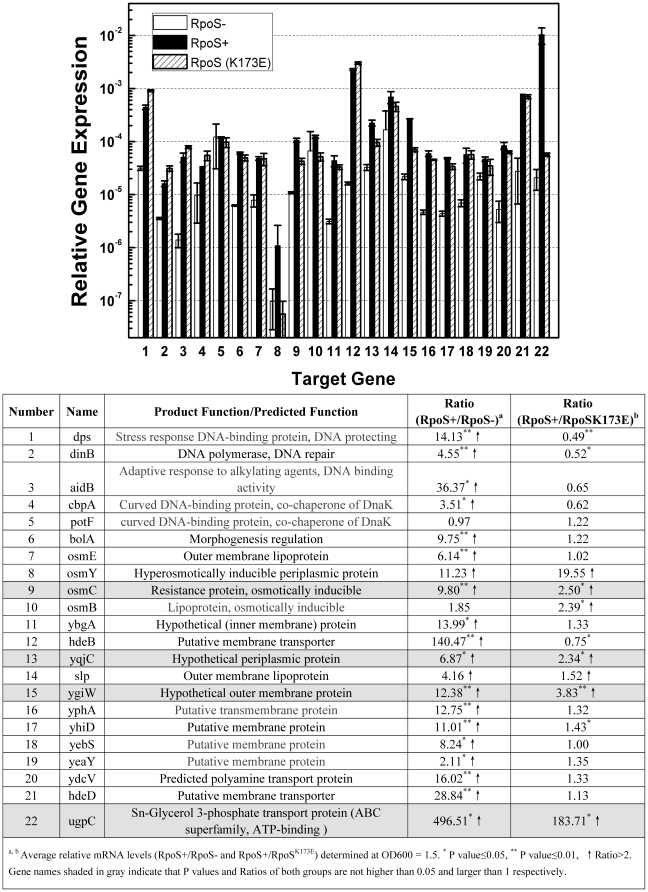
Gene expression patterns in FS20 derivatives. The expression of putative RpoS regulated genes was quantified in strains FS20-pSU (open column), FS20-pSURpoS (solid column) and FS20-pSURpoSK173E (shaded column) with Real-Time PCR with 16sRNA as a reference. Primers and names of the targeted genes were listed in a table below. Each column denotes an average of 2 samples. Error bars denote standard deviation.

Microarray data have shown that expression of a number of genes were activated by RpoS at stationary phase [16], [17], [18], [19]. To accomplish transformation, exogenous plasmid DNA needs to penetrate two membranes and be protected for plasmid establishment in cytoplasm [11], [23]. To search for RpoS regulated genes which play roles in plasmid transformation of *E. coli*, we quantified the expression of 22 of RpoS-regulated genes which are induced at stationary phase with RT-PCR. Among the 22 genes, products of 5 genes are DNA processing proteins and the other 17 genes encode membrane/periplasmic proteins. Except for *potF* and *osmB*, transcription of all the examined genes was lower in FS20-pSU than that in FS20-pSURpoS (Figure 5). Interestingly, transcription of most of these genes was not significantly reduced by K173E mutation (Figure 5). As compared to FS20-pSURpoS, transcripts of all the 5 genes for DNA processing were not significantly reduced (<2-fold or P value>0.05) in FS20-pSURpoS^K173E^ (Figure 5). Whereas among 17 genes encoding membrane/periplasmic associated proteins, the transcription of *ugpC* was more than 10-fold decreased by the mutation of K173E (Figure 5). K173E mutation also reduced the transcription of stress-inducible membrane protein encoding gene *osmC* and outer membrane encoding gene *yqjC* by 2.5 and 2.34 folds respectively (Figure 5). Besides, the transcription of *ygiW*, which is predicted to encode a periplasmic protein, was reduced 3.83-fold by K173E mutation (Figure 5). The transcript quantification data of RpoS-regulated gene suggest that RpoS may regulate plasmid transformation by controlling membrane/periplasmic proteins which could mediate dsDNA transfer across membranes.

**Figure 6:**
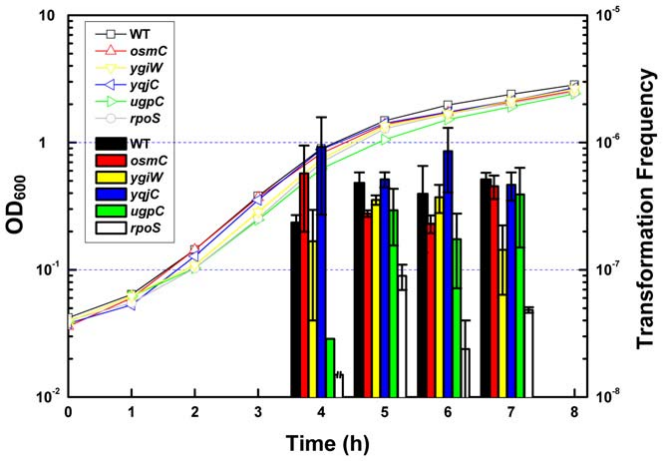
RpoS regulated genes and plasmid transformation. BW25113 and its mutant derivatives were transformed as described in Materials and Methods. Cell growths were measured by monitoring OD_600_ for 8 hours. Each point denotes an average of 2 samples. Error bars denote standard deviation.

To further screen out transformation related genes, we measured transformation frequencies in *E. coli* strains defective in RpoS-regulated genes whose transcription was reduced by both *rpoS* null mutation and K173E mutation (P value ≤0.05) by more than 2 folds. Four genes (*osmC*, *yqjC*, *ygiW* and *ugpC*) met the above criteria. We investigated their potential involvement in plasmid transformation of *E. coli* K-12 BW25113 and its mutant derivatives from Keio collection [21]. Construction of these mutants, JW1477 (*osmC*), JW2992 (*ygiW*), JW5516 (*yqjC*) and JW3415 (*ugpC*), was confirmed by PCR (see Figure S1 and Table S2). Growth curve patterns of the four mutants were similar to the wild type and *rpoS* mutant (Figure 6). Transformation of all the four mutants did not show significant difference with respect to the wild type while the *rpoS* mutant showed a noticeable transformation defect (Figure 6). The data show that single gene inactivation of *osmC*, *yqjC*, *ygiW* or *ugpC* did not affect transformation efficiency.

## Discussion

In this study, we investigated several basic questions in spontaneous plasmid transformation of *E. coli* on agar plates, which involves competence development and regulation and dsDNA transfer across two cell membranes. First, we observe that competence for plasmid transformation was induced at the beginning of stationary phase in nutrient rich medium under standard culture conditions, and that despite of the inoculum size, the induction of competence was a response to optical cell density rather than the incubation time (Figure 1). Second, the DNA uptake kinetics on agar plates shows that competent cells acquire exogenous plasmid DNA in a short time (Figure 2), suggesting the presence of a presetting DNA entry route in competent cells in liquid culture. Third, the half-order kinetics between recipient cells and transformants is detected when cell density is high on plates (Figure 3). Fourth, effects of *rpoS* inactivation and over-expression on plasmid transformation show that RpoS is involved in transformation regulation (Figure 4). Additionally, based on the transcription quantification data of RpoS-regulated genes (Figure 5), we found that transcription of 4 genes enconding membrane/periplasmic proteins was reduced by RpoS K173 mutation but inactivation of any one of them did not significantly affect plasmid transformation (Figure 6). Parameters analyzed in this and our previous studies on the plasmid DNA transfer of *E. coli* were summarized in Table S1.

### 1. The effect of inoculum size on plasmid transformation

With different inoculum sizes, the induction of competence for plasmid transformation of *E. coli* was dependent on optical cell density rather than the incubation time (Figure 1). The case in *E. coli* is different from that in *Streptococcus pneumoniae*, whose competence development is determined by the incubation time rather than cell density when the inoculum size varies [24]. The different responses to inoculum sizes in the two species are not surprising. First, *S. pneumoniae* acquires exogenous DNA through a set of proteins that are not functional in plasmid transformation of *E. coli* and therefore the regulation targets should be different in the two species [10]. Second, competence induction conditions vary largely among species even for the same set of DNA uptake machinery [24], [25], [26], [27]. Different species have evolved their own way to control the development of competence. For *E. coli*, it seems that this bacterium has evolved its own way to control competence for a different plasmid transformation mode by cell density.

### 2. DNA uptake on plates and transformation stimulator(s) in agar

To know the kinetics of DNA uptake on agar plates, we interrupted DNA uptake by plating excessive DNase I. The DNase I interruption experiment showed that the first plasmid DNA molecule should be taken up within 30 seconds and that more than one third of plasmid DNA was taken up and protected from DNase I degradation within 2 minutes after plating (Figure 2). The rapid DNA uptake on plates led us think whether a sudden oxidative stress induced DNA uptake or the physical stress during spreading pushed DNA into cells. To test the first possibility, we examined the effect of oxidative reagent H_2_O_2_ and anti-oxidant reagents Na_2_SO_3_ and NaHSO_3_ on plasmid transformation. However, we did not detect obvious effects of these oxidant and anti-oxidant reagents on transformation (see Figure S2, S3). To test whether the quick DNA uptake is an artifactual phenomenon triggered by spreading, we compared spreading of the cells on agar surface with a spreader and beads, and did not observe an obvious effect of spreading manners on transformation (see Discussion S1). If DNA uptake is mediated by a specific protein device which is synthesized after plating, such a short time would not be enough for *de novo* synthesis of a complete set of proteins for DNA uptake apparatus. More likely, the putative DNA uptake apparatus or its components might have been expressed in the liquid culture before activation by certain stimulators (e.g. divalent cations) or molecules available in a short time inside of cells (e.g. small RNA) on agar plates. Our previous work showed that plasmid transformation of *E. coli* is stimulated by agar/agarose on plates [10]. Although divalent cations chelator EGTA inhibits transformation stimulation by agar/agarose, Ca^2+^, Fe^2+^, Mg^2+^, Mn^2+^ or Zn^2+^ in agar was not identified as the transformation stimulator [10]. It remains unknown whether the transformation stimulator triggers DNA uptake within the initial several minutes or plasmid establishment in later stages.

### 3. Cell density and plasmid transformation

**Low cell density:** In both this work and our previous study, we observed that the number of transformants increased more rapid than the number of recipient cells at relative low cell density on plates (Fig. 3) [10]. A recent report indicates that cell-to-cell transformation in *E. coli* may be regulated by a pheromone [28]. In *S. pneumoniae*, it has been well established that a quorum sensing peptide regulates natural transformation through classical DNA uptake machinery [29], [30]. We tentatively propose that the increase of cell density may help accumulation of a factor that regulates plasmid transformation of *E. coli* on plates through an unknown mechanism.

**High cell density:** When cell density was relative high, the increase of the number of transformant was not quicker than the number of recipient cells (Figure 3). It is likely that when the accumulation of the putative competence factor reaches a certain concentration, its effect on plasmid transformation reaches the maximum. Based on the half-order kinetics as a function of cell density (Figure 3), we envision the possibility that cell-to-cell interaction could play a role in the formation of transformants on agar plates. The case would not be unprecedented. For example, a channel called ‘nanotube’, which connects two cells together, has been found to mediate *E. coli* cell-to-cell plasmid transfer on the solid surface [31], [32].

### 4. Regulation of plasmid transformation by RpoS

**Involvement of RpoS in plasmid transformation:** When *E. coli* cells enter the stationary growth phase, RpoS is highly expressed and many stationary phase genes are switched on [14], [15]. Our previous study showed that plasmid transformation rate is increased remarkably by statically incubating stationary phase *E. coli* in an open system [8]. Such treatment may help accumulate more RpoS. Below 30°C, the expression of RpoS is promoted by small RNA DsrA which starts accumulating at exponential phase. In the present work, we detected competence at the transition of exponential to stationary phase at 30°C. These phenomena suggest that the accumulation of RpoS may associate with the induction of competence for plasmid transformation. In this study, we confirmed a role of RpoS in spontaneous plasmid transformation of *E. coli*. Transformation frequency is reduced by inactivating *rpoS* and over-expressing *rpoS* remarkably increased transformation (Figure 4). Moreover, the K173E mutation, which alters RpoS selectivity, caused RpoS unable to promote plasmid transformation (Figure 4B), confirming both the importance of K173 to RpoS activity which had been reported [22] and a role of RpoS in plasmid transformation. K173E mutation also reduced transformation frequency in the wild type cell. It is possible that RpoS^K173E^ is able to compete with the active RpoS or other sigma factors (e.g. *rpoD*) for RNA polymerase or promoters which are important to the transcription of some genes involved in plasmid transformation. The suppression effect of K173E mutaion on plasmid transformation of the *rpoS* mutant FS20 was not obvious when cell density was high on agar plates (Figure 4B). However, we repeatedly observed a strong suppression effect of K173E mutation on plasmid transformation of the *rpoS* mutant with non-concentrated cell cultures on LB-agar plates (n>5, data not shown). It seems that when RpoS is absent, high cell density on LB-agar plates could counteract the suppression effect of K173E mutation on plasmid transformation.

**Targets for RpoS regulation?** In *Vibrio cholerae*, RpoS regulates natural competence for transformation through a quorum sensing regulator HapR which controls the transcription of a conserved DNA uptake gene [26]. Because our previous study showed that DNA uptake gene orthologs *ppdD*, *hofQ*, *gspD* and *ycaI*, together with a highly conserved transforming ssDNA processing gene *dprA*/*smf*, were not required for spontaneous plasmid transformation of *E. coli* [10], mechanisms of transformation regulation by RpoS and cell density should be different in *E. coli* and *V. cholerae*. Considering that dsDNA enters *E. coli* cells during spontaneous plasmid transformation [10], we envision that RpoS may regulate the transcription of the gene(s) which belong(s) to a new set of DNA uptake genes encoding membrane/periplasmic proteins for the passage of dsDNA and/or DNA processing genes that are required for plasmid establishment in *E. coli*. By analyzing documented microarray data, we learned that RpoS regulated the expression of at least 35 genes encoding membrane and periplasmic proteins in stationary-phase *E. coli* cells [16], [17], [18], [19]. Transcription of 22 of these genes was quantified in this work. Interestingly, transcription of most examined RpoS-regulated genes (17 out of 22) was not significantly affected by K173E mutation and of almost all these genes was higher in a strain expressing RpoS^K173E^ than in an *rpoS* mutant (Figure 5). It is possible that different promoter elements of a specific gene and their differential sensitivity to complex factors *in vivo* may complicate gene transcriptional regulation [15]. Transcription of all the five genes for DNA processing was not significantly changed by K173E mutation (Figure 5), indicating that RpoS should not regulate plasmid transformation by affecting DNA processing or plasmid establishment. By contrast, transcription of *ugpC* (encoding a glycerol ATP-binding transporter) was more than 10-fold reduced by K173E mutation (Figure 5). The other three genes, whose transcription was also significantly affected by K173E mutation (Figure 5), include *ygiW* and *yqjC* which potentially encode an outer membrane protein and a periplasmic protein respectively and an osmotically inducible gene *osmC* which encodes a resistance protein. It seems that RpoS-regulated genes encoding membrane/periplasmic proteins are more prone to be sensitive to K173E mutation. Unfortunately, our further function examination showed that single gene inactivation of *ugpC*, *ygiW*, *yqjC* or *osmC* did not significantly reduce plasmid transformation frequency (Figure 6). Possibly, RpoS regulates plasmid transformation by targeting on multiple genes which could contribute synergistically to this process. The decrease of plasmid transformation by K173E mutation may reveal that the elements in the promoters of these genes could be quite sensitive to K173 position of RpoS. The high sensitivity of these elements might help screen out genes involved in plasmid transformation of *E. coli*. By comparing global transcription profiles of competent *E. coli* cells expressing RpoS and RpoS^K173E^, together with their counterparts not expressing RpoS as a control, the scope of candidate genes for this new type of plasmid DNA transfer could be defined in further investigations.

### 5. RpoS, a potential target for controlling gene transfer?

Gene transfer often brings about new intractable pathogens, which are highly virulent and/or resistant to antibiotics. In this study, we have shown that RpoS is able to regulate gene transfer mediated by plasmid in *E. coli*. RpoS can also regulate gene transfer through chromosomal DNA transformation in *V. cholerae* [26]. Providing that RpoS is able to regulate different types of gene transfer, it is conceivable to design strategies to control gene transfer by targeting on it. For example, compounds could be designed to block K173 of RpoS or K173 mutation could be applied in the construction of bacterial vaccine strains to prevent virulent/resistant gene transfer. Besides, a group of small RNAs which regulate RpoS translation [15], could be good candidates for direct evaluation of their potential pharmaceutical effects on preventing bacterial gene transfer.

## Materials and Methods

### 1. Bacterial strains, primers and transformation of *E. coli* on plates

All of the strains and plasmids used in the present study are listed in Table 2. Primers used for cloning and Real-Time PCR (RT-PCR) are listed in Table 3. Plasmid transformation was carried out by using a developed procedure that was previously described and beads were used to spread cells on plates unless otherwise specified [8], [10]. All experiments were performed at 30°C. To prepare a pre-culture, 60 µl of the culture stock from −80°C was inoculated into 3 ml of LB broth in a glass tube and grown overnight (13∼16 hours) to a cell density of OD_600_ of ∼1.5. To prepare competent cells, unless specified otherwise, 1 ml of the pre-culture was inoculated into 100 ml of 1.5× LB broth (prepared by resolving 7.5 g yeast extract, 15 g tryptone and 7.5 g NaCl in 1 liter H_2_O) in a triangle glass flask. The culture was horizontally shaken at a speed of 150 rotations per minute. Cell growth was measured by recording the optical cell density at OD_600_. At intervals, 500 µl of the culture was precipitated and 450 µl of the supernatant was discarded. The cell pellet was resuspended in the remaining 50 µl of the supernatant and 1.5∼3.5 µg of plasmid pDsRED (final concentration, 30∼70 µg ml^−1^) was added. The mixture was immediately (<1 min) spread with glass beads onto LB plates (diameter, 6 cm) which contained 5% agar and 200 µg/ml of ampicillin, which had been air-dried at 37°C for 24∼48 hours. Transformation frequency was calculated by dividing the number of transformants by viable counts.

**Table 3:** Primers used in this study.

Name	Primer Forward	Primer Reverse	Reference
*rpoS*	c**TCTAGA**tggtccgggaacaacaagaagtta *	c**GGGATC**ccttaattacctgtgtgcgtat	[36], [37]
*rpoS* K173E	cgattcacatcgta**G**aggagctgaacgtt &	aacgttcagctcct**C**tacgatgtgaatcg	[38]
*dps*	tgaaaagttacccgctggac	ggtcaggatatctgcggtgt	This study
*dinB*	ggctgtatccggaacttgaa	ggtggtttgctgaaaatcgt	This study
*aidB*	tgttgcgcgttctcaataag	tgctgctgtaaacgacgaac	This study
*cbpA*	ggcgatctgtatgcggtact	tttcccccaatctttacgtg	This study
*potF*	aattaatggccgggagtacc	agttccgggtcgagattctt	This study
*bolA*	cggctctgaaagccatttta	cagcgcatgaacggtagtag	This study
*osmE*	cctgtggtgaaagacgtcaa	tcacgttgacccaggatgta	This study
*osmY*	aacacaaaatttgcccgttc	cacgttgtcggctttattga	This study
*osmC*	gggaacagtatccaccgaga	aaagcgccattgagaaacat	This study
*osmB*	cggctgttctggcaattact	agtactgcaccgcctaatgc	This study
*ybgA*	cgctgatgcgtatcaaacac	cctgaatggcgcaagttaat	This study
*hdeB*	cactggtgaacgcacaatct	tttcttcatgcagcatccac	This study
*yqjC*	gtgccggtagttatgccact	gggctttattcagaccgtca	This study
*slp*	gggtgaacaacctggcttta	atgcaccatagccgtaatcc	This study
*ygiW*	tcgttgaacgcatctctgac	cgacttcaccctgaatctcaa	This study
*yphA*	ccaatatatggcctcgttgg	gtgaaaaagccaagcacgat	This study
*yhiD*	ttcgctgattggtttgttga	tcagccactcaatgctgttc	This study
*yebS*	gttactgcggatggtgacct	cagccggtcccatagtaaaa	This study
*yeaY*	gtggatttccgtggacaact	aacccgttacttgcatcacc	This study
*ydcV*	ggcagcacagcgtagtgata	ttttgccgaaaaagtctcgt	This study
*hdeD*	gtttatcgtcgggttgctgt	ttatgactgcggttgctgaa	This study
*ugpC*	ataacgaaggcacgcatttc	cgaatgccgagagtcatttt	This study
*osmC* (CHK)	cctgcttatcctcgtgctgt	taacaacgcatcaggcattc	This study
*ygiW* (CHK)	acgcgctctgtacagcacta	caacatcgccacaggtattg	This study
*yqjC* (CHK)	cagtggctgacccggtta	ccagcgtatcggaaagggat	This study
*ugpC* (CHK)	tgttaacgcttatccctccg	gattgacgccagggtgtttt	This study
*rpoS* (CHK)	ggtccgggaacaacaagaagtta	cggattcttaattacctggtgcgtat	This study

* capital letters in bold indicate the restriction enzyme recognizing sequence;

& capital letter in bold with underline indicates the position of nucleoside for mutation.

To investigate the effect of inoculum size on plasmid transformation, 0.01, 0.1 and 1 ml of the overnight grown culture were inoculated to three glass flasks. To investigate the effect of cell density on plasmid transformation, cell pellets from 100 µl, 200 µl, 500 µl and 1000 µl of the culture were resuspended in 100 µl of the supernatant and mixed with 6.6 µg of pDsRED, followed by spreading 50 µl of the mixture onto a selective plate. To know whether *E. coli* was transformed prior to plating, DNase I (∼2 mg/ml, final concentration) was added to the mixture of the culture and pDsRed which had been co-incubated for 10 minutes, then STOP solution (Promega Corp.) was added after 10 or 20 minutes of DNase I treatment. The control was treated in the same manner except that DNase I was not added. To measure the kinetics of DNA uptake on plates, 0 min, 0.5 min, 1 min, 2 min, 5 min and 10 min after plating the mixture of competent cells, 20 µl of 25 mg/ml DNase I was spread on the plate.

### 2. Recombinant plasmids and strains construction

A DNA fragment containing the *rpoS* gene together with its 205 bp upstream and 127 bp downstream nucleotides, was amplified from the *E. coli* ZK126 genome DNA. Primers for cloning the DNA fragment are listed in Table 3. The purified PCR product was cut by *Xba*I and *BamH*I and then ligated to the cloning vector pUC18 to form pUCRpoS. The PCR-derived part in pUCRpoS was sequenced. To construct a plasmid expressing *rpoS* and compatible with the transformation donor plasmid pDsRED, the fragment with the correct *rpoS* sequence was cut from pUCRpoS and ligated to pCHAP3100 with blunt ends (digested by *EcoR*I and *Hind*III to delete the *ppdD* gene and then treated with mungbean nuclease) to construct pSURpoS. The empty vector pSU was constructed by deleting the *rpoS* gene in pSURpoS. The two plasmids, pSURpoS and pSU, were transformed to FS20 and MC4100 to create strains FS20-pSU, FS20-pSURpoS, MC4100-pSU and MC4100-pSURpoS.

### 3. Site-directed mutagenesis of K173 of RpoS

With plasmid pSURpoS DNA as the template, the whole-length plasmid DNA was amplified by two complementary primers K173E-1 and K173E-2 for mutagenesis (Table 3). The PCR product was digested by *Dpn*I to eliminate template pSURpoS DNA and transformed to *E. coli* ZK126. The PCR-derived parts in the resulting RpoS mutant plasmids were sequenced to confirm the desired mutation. The plasmid with an *rpoS* derivative gene encoding a protein with K173E mutation was named pSURpoS^K173E^, which was transformed into FS20 and MC4100 to create the strain FS20-pSURpoS^K173E^ and MC4100-pSURpoS^K173E^.

### 4. RNA isolation and real-time PCR

When cells grew to the optical density of OD_600_ of 1.5 in 100 ml of 1.5× LB broth, RNA was isolated from FS20-pSU, FS20-pSURpoS and FS20-pSURpoS^K173E^ with a protocol provided by the RNA isolation kit (Tiangen Company) and reverse transcribed to cDNA. RT PCR was performed with the CFX-96 sequence detection system (Bio-Rad, USA). SYBR Green Real-Time PCR Mix (Bio-Rad) was used with a primer concentrations of 50 nM. Primer sequences were listed in Table 3. Reaction conditions were 95°C for 3 minutes, followed by 40 cycles of 95°C for 10 seconds and 60°C for 30 seconds. Duplicate PCRs were run for each cDNA sample. 16SrRNA was selected as the reference against which the starting quantity of RNA was normalized. The threshold cycles were calculated with the Bio-Rad CFX-96 manager software. The relative expression of genes were calculated by the formula 2^(ΔCt Taget−ΔCt Reference)^ [33].

### 5. Examination of mutants from Keio collection

Control PCR experiments confirmed the loss of wild-type gene fragments in the mutant strains from Keio collection [21] and their replacement by a fragment, the size of which was fully consistent with that predicted from simple insertion of antibiotic-resistance gene cassette (see Figure S1 and Table S2).

## Supporting Information

Figure S1 **Examination of the structure of** ***E. coli*** **mutants tested in** **Figure 6** **(see accompanying Table S2).** Analysis of PCR fragments for confirmation of the structure of *E. coli* mutant strains (Primers used were listed in Table 2). From left to right, each pair of lanes compares wildtype and mutant structures, as follows. Lanes: 1, 4 and 13, DD Marker 2, *ugpC* mutant; 3, wide type 5, *rpoS* mutant; 6, wide type 7, *yqjC* mutant; 8, wide type 9, *ygiW* mutant; 10, wide type 11, *osmY* mutant; 12, wide type.
(TIF)

Figure S2 **Effect of H_2_O_2_ on plasmid transformation.** H_2_O_2_ was added either at OD_600_ = 0.1 (A) or OD_600_ = 1.5 (B). Plasmid transformation was measured either 4, 5 and 6 hours (A) or 10 minutes (B) after the addition of H_2_O_2_. Transformation frequency was calculated by dividing the number of transformants per ml by the number of viable counts per ml. Each sample was performed in duplicate. Error bars denote standard deviation.
(TIF)

Figure S3 **Effect of anti-oxidant agents on plasmid transformation.** Anti-oxidant agents Na_2_SO_3_ or NaHSO_3_ were added to the liquid culture prior to plating (A) or the solid agar plates for plasmid transformation (B). Transformation frequency was calculated by dividing the number of transformants per ml by the number of viable counts per ml. Each sample was performed in duplicate. Error bars denote standard deviation.
(TIF)

Table S1 **Summary of examined factors in plasmid transformation of** ***E. coli*** **.**
(DOC)

Table S2 **Examination of the structure of** ***E. coli*** **mutant strains used in this work.**
(DOC)

Discussion S1 **Plasmid transformation and stresses.**
(DOC)
