# Supplementary figures and images for: RpoS Regulates a Novel Type of Plasmid DNA Transfer in Escherichia coli
Source: PLoS One. 2012 Mar 16;7(3):e33514. doi: 10.1371/journal.pone.0033514 (PMC3306417; doi:10.1371/journal.pone.0033514)

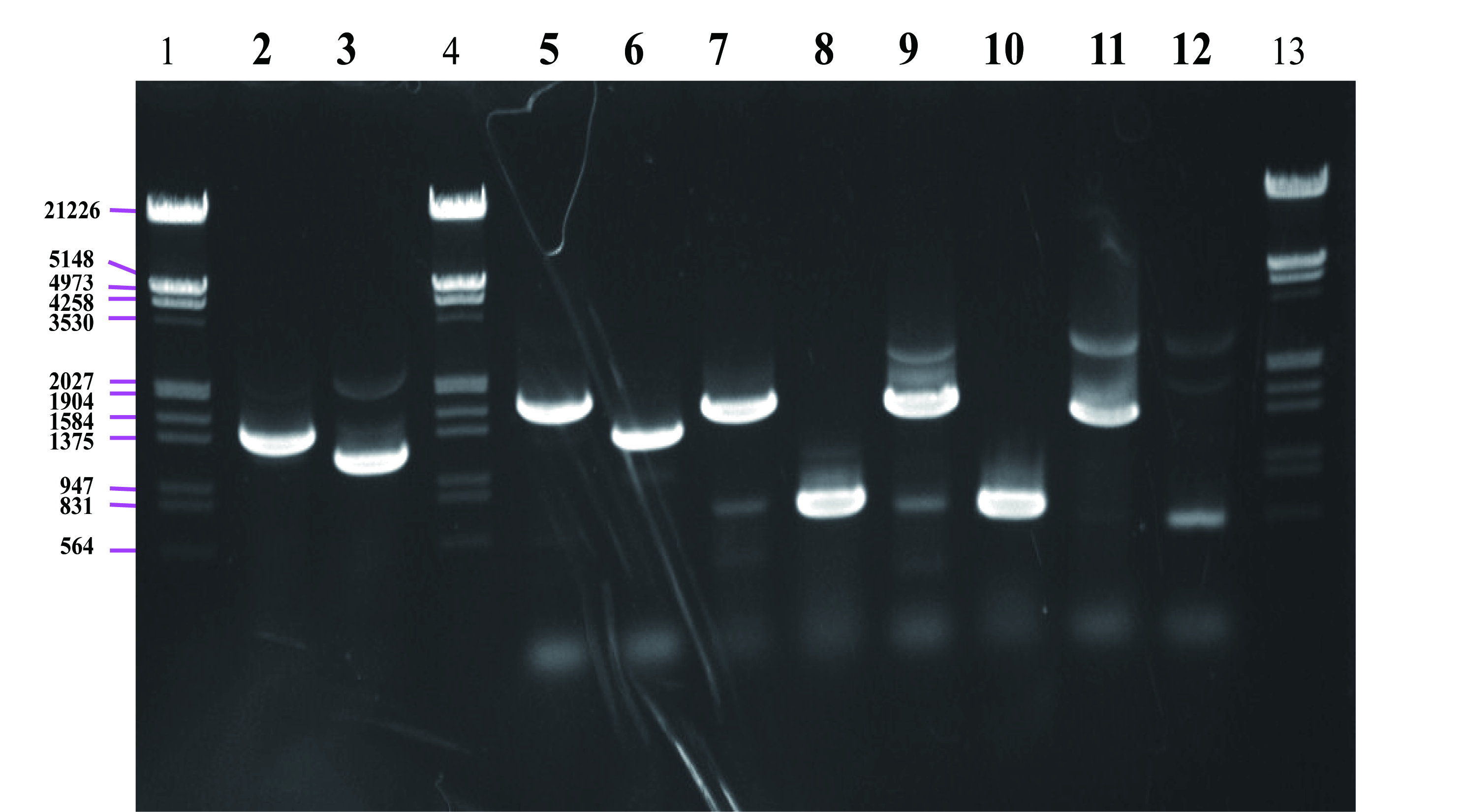

Supplement: Figure S1 — Examination of the structure of E. coli mutants tested in Figure 6 (see accompanying Table S2). Analysis of PCR fragments for confirmation of the structure of E. coli mutant strains (Primers used were listed in Table 2). From left to right, each pair of lanes compares wildtype and mutant structures, as follows. Lanes: 1, 4 and 13, DD Marker 2, ugpC mutant; 3, wide type 5, rpoS mutant; 6, wide type 7, yqjC mutant; 8, wide type 9, ygiW mutant; 10, wide type 11, osmY mutant; 12, wide type. (TIF) [file pone.0033514.s001.tif]

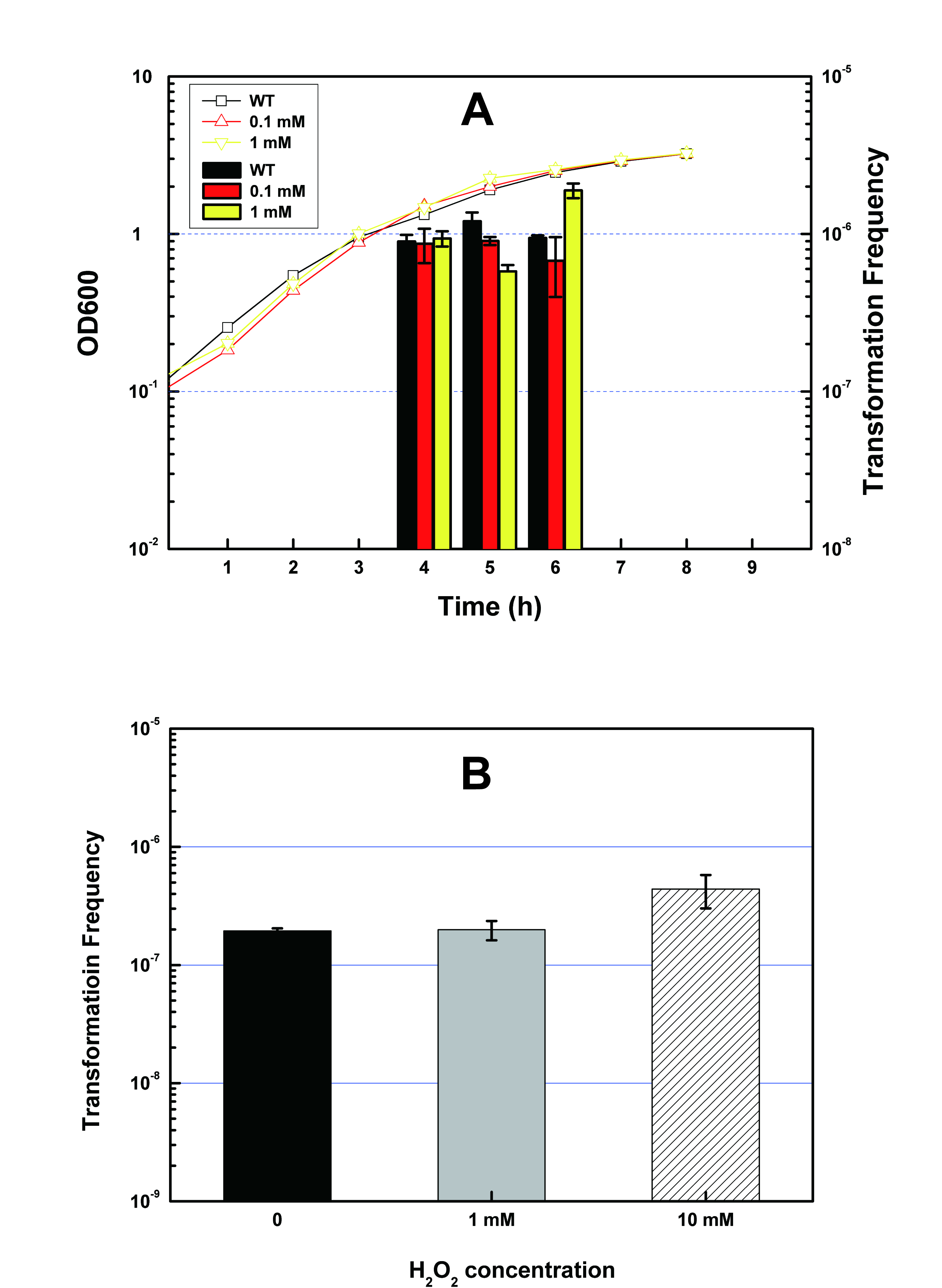

Supplement: Figure S2 — Effect of H2O2 on plasmid transformation. H2O2 was added either at OD600 = 0.1 (A) or OD600 = 1.5 (B). Plasmid transformation was measured either 4, 5 and 6 hours (A) or 10 minutes (B) after the addition of H2O2. Transformation frequency was calculated by dividing the number of transformants per ml by the number of viable counts per ml. Each sample was performed in duplicate. Error bars denote standard deviation. (TIF) [file pone.0033514.s002.tif]

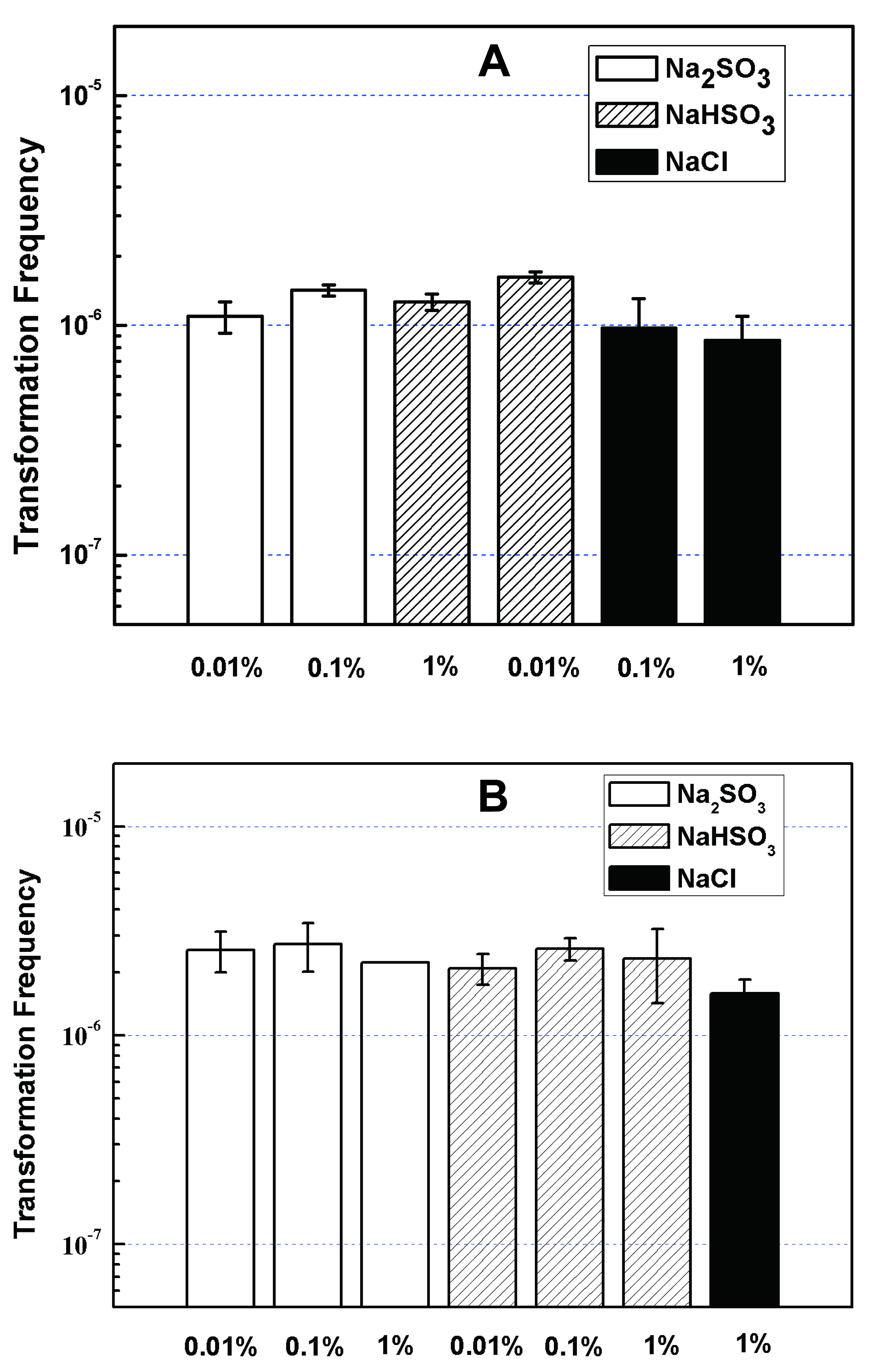

Supplement: Figure S3 — Effect of anti-oxidant agents on plasmid transformation. Anti-oxidant agents Na2SO3 or NaHSO3 were added to the liquid culture prior to plating (A) or the solid agar plates for plasmid transformation (B). Transformation frequency was calculated by dividing the number of transformants per ml by the number of viable counts per ml. Each sample was performed in duplicate. Error bars denote standard deviation. (TIF) [file pone.0033514.s003.tif]
