# Supplementary material for: RpoS Regulates a Novel Type of Plasmid DNA Transfer in Escherichia coli
Source: PLoS One. 2012 Mar 16;7(3):e33514. doi: 10.1371/journal.pone.0033514 (PMC3306417; doi:10.1371/journal.pone.0033514)
Supplement: Table S1 — Summary of examined factors in plasmid transformation of E. coli . (DOC) [file pone.0033514.s004.doc]

**Table S1. Summary of examined factors in plasmid transformation of *E. coli*.**

| **Factors** | **Effects** | **Reference** |
| --- | --- | --- |
| Stress response genes | | |
| *rpoS* | ↑^a^ | Figure 4 |
| *osmC* (RpoS regulated) | - ^c^ | Figure 6 |
| *ygiW* (RpoS regulated) | - | Figure 6 |
| *yqjC* (RpoS regulated) | - | Figure 6 |
| *ugpC* (RpoS regulated) | - | Figure 6 |
| Oxidative stresses | | |
| Oxygen | - | [1] |
| Nitrogen | - | [1] |
| Na_2_SO_3_ (anti-oxidative agent) | - | Figure S3 |
| NaHSO_3_ (anti-oxidative agent) | - | Figure S3 |
| H_2_O_2_ | - | Figure S2 |
| Physical stress | | |
| Spreading with beads | - | This study |
| Spreading with a spreader | - | This study |
| Cell density | | |
| Low | Non-linear kinetics | [1] |
| High | Half-order kinetics | Figure 3 |
| Growth phase | Stationary phase | [1] & Figure 1 |
| DNase I | Sensitive | [1] & Table 3 |
| Agar/agarose |  |  |
| EGTA | ↓^b^ | [2] |
| concentration | ↑ | [2] |
| Divalent cations | | |
| Ca^2+^ | - | [2] |
| Mg^2+^ | - | [2] |
| Mn^2+^ | - | [2] |
| Fe^2+^ | - | [2] |
| Zn^2+^ | - | [2] |
| DNA uptake gene orthologs | | |
| *ycaI* (rec-2/comEC homolog) | - | [2] |
| *hofQ*/*gspD* | - | [2] |
| *ppdD* | - | [2] |
| *dprA* | - | [2] |
| DNA dosage | One-order kinetics | [1] |

^a^, ↑ negative effect; ^b^, ↓ positive effect; ^c^ - no effect

**References**

1. Sun D, Zhang Y, Mei Y, Jiang H, Xie Z, et al. (2006) *Escherichia coli* is naturally transformable in a novel transformation system. FEMS Microbiol Lett 265: 249-255.

2. Sun D, Zhang X, Wang L, Prudhomme M, Xie Z, et al. (2009) Transforming DNA uptake gene orthologs do not mediate spontaneous plasmid transformation in *Escherichia coli*. J Bacteriol 191: 713-719.
