# Supplementary material for: RpoS Regulates a Novel Type of Plasmid DNA Transfer in Escherichia coli
Source: PLoS One. 2012 Mar 16;7(3):e33514. doi: 10.1371/journal.pone.0033514 (PMC3306417; doi:10.1371/journal.pone.0033514)
Supplement: Table S2 — Examination of the structure of E. coli mutant strains used in this work. (DOC) [file pone.0033514.s005.doc]

**Table S2. Examination of the structure of *E. coli* mutant strains used in this work**

| **Lane^a^** | **Strain^b^** | **Primer pair^b^** | **Size^c^ (kb)** |
| --- | --- | --- | --- |
| **1** | Double Digest Marker (DD Marker, Fermentas Corp.) | | |
| **2** | JW3415 (*ΔugpC*) | *ugpC* (CHK) | 1.49 kb |
| **3** | BW25113 | *ugpC* (CHK) | 1.23 kb |
| **4** | DD Marker | | |
| **5** | JW5437 (*ΔrpoS*) | *rpoS* (CHK) | 1.78 kb |
| **6** | BW25113 | *rpoS* (CHK) | 1.33 kb |
| **7** | JW5516 (*ΔyqjC*) | *yqjC* (CHK) | 1.62 kb |
| **8** | BW25113 | *yqjC* (CHK) | 0.66 kb |
| **9** | JW2992 (*ΔygiW*) | *ygiW* (CHK) | 1.54 kb |
| **10** | BW25113 | *ygiW* (CHK) | 0.61 kb |
| **11** | JW1447 (*ΔosmC*) | *osmC* (CHK) | 1.45 kb |
| **12** | BW25113 | *osmC* (CHK) | 0.55 kb |
| **13** | DD Marker | | |

^a^ Lane numbers refer to Figure S1

^b^ Strains and PCR primers are listed in Table 2

^c^ Sizes predicted on the basis of available DNA sequence information for PCR fragments generated with primer pairs indicated in the third column. When the predicted sizes of wildtype and mutant PCR fragments were too close, the fragments were discriminated through digestion with the indicated restriction enzyme

Sizes of bands observed on the gels shown in Figure S1 are in good agreement with the prediction.
