# Supplementary material for: RpoS Regulates a Novel Type of Plasmid DNA Transfer in Escherichia coli
Source: PLoS One. 2012 Mar 16;7(3):e33514. doi: 10.1371/journal.pone.0033514 (PMC3306417; doi:10.1371/journal.pone.0033514)
Supplement: Discussion S1 — Plasmid transformation and stresses. (DOC) [file pone.0033514.s006.doc]

**Supporting information**

**Discussion S1: plasmid transformation and stresses.**

Examined factors which potentially affect plasmid transformation have been summarized in Table S1. Compared with traditional natural and artificial transformation, this new type of plasmid transformation shows several remarkable characters. First, plasmid transformation occurs exclusively on agar plates on which agar/agarose concentration strongly promotes transformation [1] [2] (Figure 2). Second, transforming DNA uptake orthologs do not mediate DNA transfer during plasmid transformation on plates [2]. Third, cell density affects plasmid transformation on plates [2] (Figure 3). Fourth, RpoS, the general stress response regulator, is involved in plasmid transformation on plates (Figure 4).

In this study, we have examined potential roles of RpoS regulated genes encoding membrane/periplasmic proteins in plasmid transformation (Figure 6). The failure of screening out any RpoS regulated genes involved in plasmid transformation raised the possibility that the plasmid transformation promotion activity of RpoS might be an indirect effect of external stresses which were introduced during plating. Hereafter, we evaluated several stresses in plasmid transformation of *E. coli* on plates. To know whether physical stress pushes plasmid DNA into cells on agar plates, we compared effects of spreading with a spreader and beads on plasmid transformation. No significant difference in plasmid transformation was detected between the two different spreading ways (144 ± 50 cfu/ml with beads and 222 ± 26 with the spreader). We also compared the effect of physical stress on plasmid transformation by spreading with beads for a serial time (30, 45, 60, 75 and 90 seconds) and did not observe significant difference either (Data not shown). Therefore, physical stress by spreading is not likely a trigger of plasmid transformation on plates. To evaluate the effect of oxidative stress on plasmid transformation, we added the oxidative agent H_2_O_2_ in the liquid culture. At the non-lethal concentration (≤ 1 mM), plasmid transformation was not significantly affected by the addition of H_2_O_2_ several hours or 10 minutes before plating (Figure S2). At the lethal concentration (10 mM), transformation frequency seemed slightly increased (Figure S2B). However, considering the effect of cell density on plasmid transformation (Figure 3), the slight increase of transformation frequency by the addition of 10 mM H_2_O_2_ could be an indirect effect of the decrease of viable cell density. To know whether anti-oxidation affects plasmid transformation, we added anti-oxidative agents Na_2_SO_3_ and NaHSO_3_ either to the culture to be plated or into LB agar plates while they had been prepared. The addition of these agents did not significantly (P value > 0.05) promoted plasmid transformation (Figure S3), indicating that plasmid transformation was not induced by anti-oxidative stresses. Together, physical stresses and oxidative/anti-oxidative stresses seemed not affect this new type of plasmid transformation. Our study can not exclude the possibility that plasmid transformation may be induced by other stresses on plates.

**References**

1. Sun D, Zhang Y, Mei Y, Jiang H, Xie Z, et al. (2006) *Escherichia coli* is naturally transformable in a novel transformation system. FEMS Microbiol Lett 265: 249-255.

2. Sun D, Zhang X, Wang L, Prudhomme M, Xie Z, et al. (2009) Transforming DNA uptake gene orthologs do not mediate spontaneous plasmid transformation in *Escherichia coli*. J Bacteriol 191: 713-719.
